# Electronic Implementation of Integrated End-of-life Care: A Local Approach

**DOI:** 10.5334/ijic.2507

**Published:** 2017-06-20

**Authors:** Daniel Schlieper, Christiane Altreuther, Manuela Schallenburger, Martin Neukirchen, Andrea Schmitz, Christian Schulz-Quach

**Affiliations:** 1 Interdisciplinary Centre for Palliative Medicine, Medical Faculty, Heinrich Heine University, Düsseldorf, DE; 2 Interdisciplinary Centre for Palliative Medicine and Department of Anesthesiology, Medical Faculty, Heinrich Heine University, Düsseldorf, DE; 3 LVR Clinic of Psychiatry, Psychosomatic and Psychotherapy for children and adolescence, Viersen, DE; 4 Institute of Psychiatry, Psychology and Neuroscience (IoPPN), King’s College London, UK

**Keywords:** electronic medical record, integrated care, Liverpool Care Pathway, palliative care, patient centred care, hospital

## Abstract

**Introduction:**

The Liverpool Care Pathway for the Dying Patient is an instrument to deliver integrated care for patients in their last hours of life. Originally a paper-based system, this study investigates the feasibility of an electronic version.

**Methods:**

An electronic Liverpool Care Pathway was implemented in a specialized palliative care unit of a German university hospital. Its use is exemplified by means of auditing and analysis of the proportion of recorded items.

**Results:**

In the years 2013 and 2014 the electronic Liverpool Care Pathway was used for the care of 159 patients. The uptake of the instrument was high (67%). Most items were recorded. Apart from a high usability, the fast data retrieval allows fast analysis for auditing and research.

**Conclusions and discussion:**

The electronic instrument is feasible in a computerized ward and has strong advantages for retrospective analysis.

**Trial registration:**

Internal Clinical Trial Register of the Medical Faculty, Heinrich Heine University Düsseldorf, No. 2015124683 (7 December 2015).

## Introduction

Tools for efficient collaboration of the teams are important for the best quality of patient centred palliative care [1,2,3,4]. The Liverpool Care Pathway for the Dying Patient (LCP) is such a tool to deliver integrated care for the dying [5,6]. It is an evidence-based educational and quality assurance tool to translate best-practice care from hospices into hospitals and other settings [6]. Its aims are good and transparent communication within the team and with the patients and their relatives and carers [7]. The LCP calls for regular multi-professional team assessments to confirm the diagnosis that the patient could be dying [8]. Additionally, the LCP documents the assessment of a number of goals of care to ensure coherent delivery of care, including a multi-professional team assessment of the initial care situation, the assessment of the ongoing care and the assessment of the care after death [8]. The LCP includes comprehensive recording of achievements or variants of the goals of care and has the capacity of replacing any other medical record [8]. In interviews with critical care practitioners the LCP was perceived as a useful tool for the seamless coordination of care [9].

The LCP is translated into a number of languages and is used in 22 countries [10]. Weak infrastructure [11] and poor implementation in some locations in Great Britain led to the recommendation to phase out the LCP in the UK [12,13,14]. However, it is widely accepted that a proper implementation of the LCP helps to achieve a good death [12,15,16,17]. Careful multi-professional assessments on a regular basis, as required by the LCP [18], prevent the diagnosis of dying from becoming self-fulfilling. Instead, it is common to discontinue LCP care because a number of patients recover [19].

The original LCP is a paper document [8]. The lack of an electronic version can be a barrier to its implementation [20]. For audit and research, the data need to be fed into a computer [21]. To overcome this technical barrier, audits typically use a small sample of the patient data, e.g., 30 patients per hospital [22].

This study is based on the hypothesis that an electronic version of the LCP would further improve clinical integration of care for the dying in a hospital. The aim was to facilitate the auditing of the care practice for a continuous quality improvement using a cycle of audit, reflection and education [22,23]. This cycling ensures the lasting implementation of the LCP as a quality assurance tool in end-of-life-care [24].

With an electronic LCP, all data are readily available in a database and comparative audits are much simpler. Furthermore, the data of the complete patient group are available for retrospective research analysis. This article describes the use of an electronic version of the LCP in a university hospital and discusses the problems encountered during its implementation and use.

## Description of the care practice

This study was approved by the ethics board of the Medical Faculty of Heinrich Heine University Düsseldorf (protocol number 5351R, approved 22.12.2015).

The electronic version of an end-of-life care plan, i.e., the Liverpool Care Pathway [8] was implemented in a specialized palliative care unit (SPCU) in a university hospital in Germany. The SPCU team comprised 16 palliative care nurses, six physicians (specialized in anesthesiology, psychosomatic medicine and hemato-oncology), two psycho-oncologists/art therapists, two physiotherapists, a psychologist and a social worker. In addition, the team was supported by clergy, volunteers and others. The SPCU team provides care on the ward (8 single-bed rooms) and supports the care of palliative patients on other acute wards (approximately 400 patients/year). The team had access to 15 shared desktop computers. All computers were connected to a central hospital information system (Medico, Cerner, North Kansas City, MO, USA). A ward trolley with a dedicated computer offers easy access to the electronic medical records during daily ward rounds.

Standard documentation of care includes use of scores for a wide number of symptoms such as pain, dyspnoea, nausea, restlessness and others [19]. Patients are scored routinely every 8 hours. All interventions are documented in an electronic system that allows entering free text. This documentation system is open to all members of the team with the exception of clergy and volunteers. Patients were diagnosed as dying if all four criteria by Ellershaw and Ward [5] were met according to a multi-professional team assessment: The patient becomes bedbound, is semi-comatose, is only able to take sips of fluid and is unable to take oral drugs. Additionally, a decreased functional status over time and no acute reversible reason for their decline was considered [19].

The electronic LCP was implemented in seven steps. A multi-professional expert panel (comprising specialist nurses, physicians and information technology specialists) was appointed to oversee the whole process. The SPCU was chosen as pilot site. The first version was a verbatim implementation of the paper version of LCP version 12 in German (a booklet with 16 pages) [8,25]. The user interface was seamlessly integrated into the electronic medical record system of the hospital. This version was assessed by a review panel (two palliative care nurses and two physicians). Next, the electronic LCP was linked to the hospital information system to maximise integration. Thus, the patient’s data such as name, date of birth and contact details of relatives or carer are automatically entered into the electronic LCP. Software errors and problems with the user interface were identified and corrected in a test phase with dummy patients. After another evaluation by the review panel, two dying patients were included in the electronic LCP. With the approval of the expert panel, the electronic LCP was disseminated on the SPCU in November 2012. The Medico module (in German) is available by request from the authors.

Data collection for this study started two months after dissemination of the electronic LCP. This way, the SPCU team had time to become fully proficient in its use. Because all data are electronically stored and linked to the patients’ electronic medical records, the retrieval for auditing is a simple query in Structured Query Language by the database administrator. Equally simple is the data retrieval for observational studies such as length of survival during LCP care [19]. To demonstrate that it is feasible to perform a retrospective audit on the complete group of LCP patients rather than a small sample, the most important results of this audit are shown here.

The audit analysed the outcomes of 159 patients who received LCP care between January 2013 and December 2014. These patients formed a cohort in a retrospective observational study published elsewhere [19]. During this period of time, a total of 382 patients were treated on the SPCU with an average re-admission rate of 1.17. The average age of the patients was 67.3 years (range 28–97). In total, 239 patients died. Eighty patients (33%) died suddenly without the diagnosis of dying by the multi-professional team and did not receive LCP care [19].

**Figure 1:**
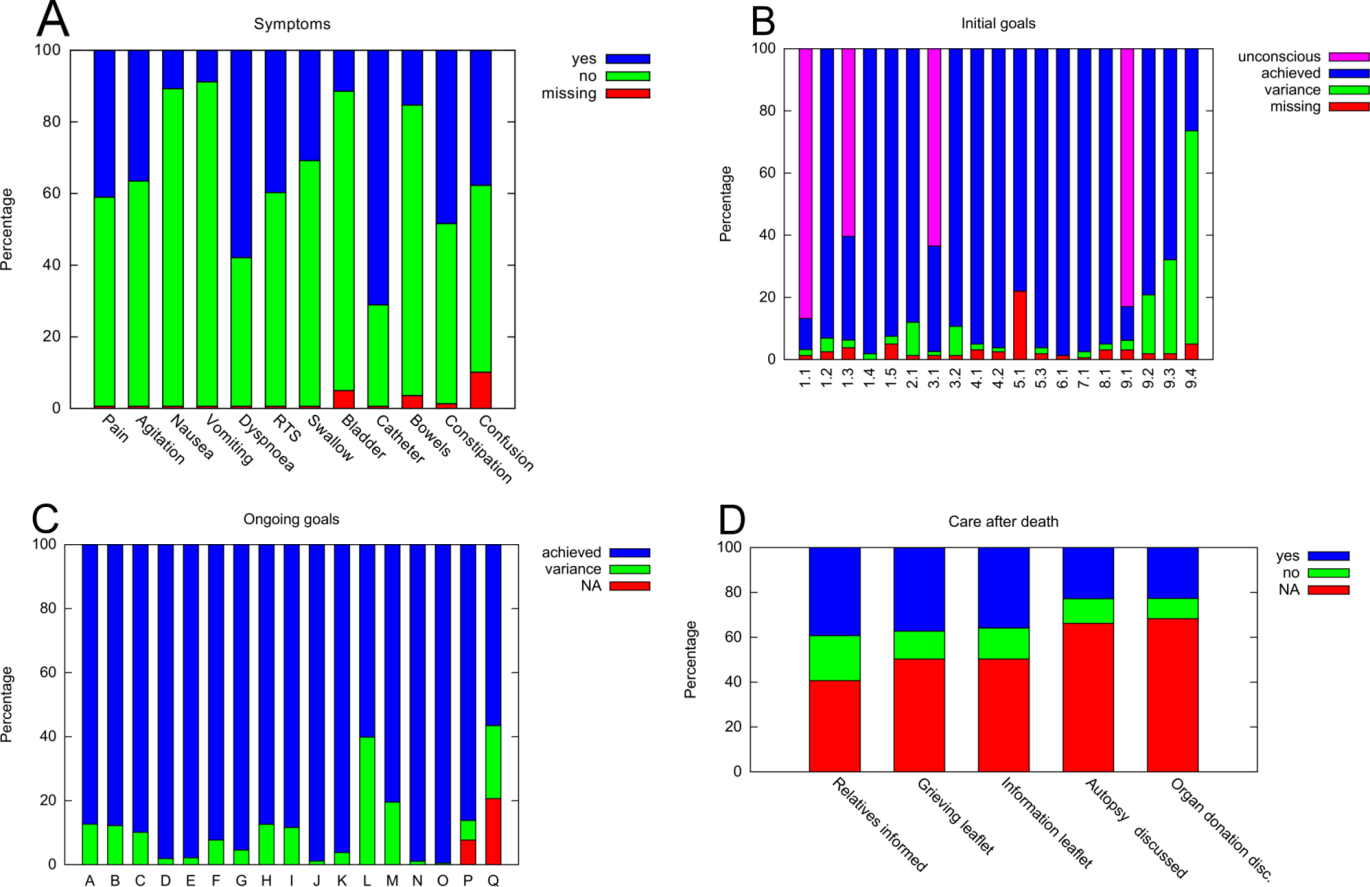
Results from auditing of 159 cases. **(A)** Initial assessment of symptoms (RTS, respiratory tract secretions). **(B)** Initial goals according to the LCP manual [8]. The areas comprise communication (goals 1.1–1.5), facilities (goal 2.1), spirituality (goals 3.1 and 3.2), medication (goals 4.1 and 4.2), current interventions (goals 5.1 and 5.3), nutrition and hydration (goals 6.1 and 7.1), skin care (goal 8.1) and explanation of the plan of care (goals 9.1–9.4) as follows: goal 1.1, *The patient is able to take a full and active part in communication;* goal 1.2, *The relative or carer is able to take a full and active part in communication;* goal 1.3, *The patient is aware that they are dying;* goal 1.4, *The relative or carer is aware that the patient is dying;* goal 1.5, *The clinical team have up to date contact information for the relative or carer;* goal 2.1, *The relative or carer has had a full explanation of the facilities available for them and a facilities leaflet has been given;* goal 3.1, *The patient is given the opportunity to discuss what is important to them at this time, e. g., their wishes, feelings, faith, beliefs, values;* goal 3.2, *The relative or carer is given the opportunity to discuss what is important to them at this time, e.g., their wishes, feelings, faith, beliefs, values;* goal 4.1, *The patient has medication prescribed on a* pro re nata *basis for all of the following five symptoms which may develop in the last hours or days of life: pain, agitation, respiratory tract secretions, nausea/vomiting, dyspnoea;* goal 4.2, *Equipment is available for the patient to support a continuous subcutaneous infusion of medication where required;* goal 5.1, *The patient’s need for current interventions has been reviewed by the multidisciplinary team;* goal 5.3, *Implantable cardioverter defibrillator is deactivated;* goal 6.1, *The need for clinically assisted (artificial) nutrition is reviewed by the multidisciplinary team;* goal 7.1, *The need for clinically assisted (artificial) hydration is reviewed by the multidisciplinary team;* goal 8.1, *The patient’s skin integrity is assessed;* goal 9.1, *A full explanation of the current plan of care (LCP) is given to the patient;* goal 9.2, *A full explanation of the current plan of care (LCP) is given to the relative or carer;* goal 9.3, *The LCP coping with dying leaflet or equivalent is given to the relative or carer;* goal 9.4, *The patient’s primary health care team/GP practice is notified that the patient is dying*. NB: goal 5.2 (*The patient has a Do Not Attempt Cardiopulmonary Resuscitation Order in place*) was a requirement for all patients on the SPCU and therefore not recorded. **(C)** Achieved goals and variances during ongoing assessments (4 hours per visit). The letters indicate the goals according to the LCP manual [8]. Goal A, *The patient does not have pain;* goal B, *The patient is not agitated;* goal C, *The patient does not have respiratory tract secretions;* goal D, *The patient does not have nausea;* goal E, *The patient is not vomiting;* goal F, *The patient is not breathless;* goal G, *The patient does not have urinary problems;* goal H, *The patient does not have bowel problems;* goal I, *The patient does not have other symptoms;* goal J, *The patient’s comfort and safety regarding the administration of medication is maintained;* goal K, *The patient receives fluids to support their individual needs;* goal L, *The patient’s mouth is moist and clean;* goal M, *The patient’s skin integrity is maintained;* goal N, *The patient’s personal hygiene needs are met;* goal O, *The patient receives their care in a physical environment adjusted to support their individual needs;* goal P, *The patient’s psychological well-being is maintained;* goal Q, *The well-being of the relative or carer attending the patient is maintained.* NA, not applicable. **(D)** Care after death (disc., discussed). NB: Some items of this section, such as the time of death, are recorded in other sections of the electronic hospital information system and thus not included here.

Figure 1 shows the main results of this audit at four different stages of care: symptom recording (Figure 1A), initial assessment (Figure 1B), ongoing care (Figure 1C) and care after death (Figure 1D).

## Discussion

The computerized ward offers a number of opportunities for the improvement of hospital care. Electronic recording improves the rate of understandable records and also results in more recorded details [26]. During integration of the LCP documentation into the hospital information system, great attention was paid to obtain an electronic version that reflects the paper version as closely as possible. This way, the electronic document is consistent with the LCP used internationally. As a side effect, the transition from paper LCP to the electronic version is straight forward, even for those members of the team who are less computer experienced. Nurses need good information technology competencies for end-of-life care planning [27], as do all other members of the multi-professional care team.

Horey et al. report that the lack of an electronic version was a barrier to the implementation of a modified version of the LCP [20]. The uptake of LCP care is 67% in this study. This proportion of patients dying on LCP care is in the same range as the average of 47.4% as reported by Stocker and Close [28]. The high uptake may indicate that the electronic LCP documentation is convenient. The high number of the recorded items (achieved goals or recorded variances) demonstrates the feasibility of the electronic LCP. As was proposed earlier [23], the data collection in the course of usual care results in a high reliability, completeness and data quality. The items are documented to a high degree of completeness. The last section, *Care after death*, is not as heavily used as the rest of the LCP. The relevant items are recorded elsewhere on the hospital information system. Here may be room for improvements of the LCP document, e. g., by automatic retrieval of the relevant data from the hospital information system.

An important part of the LCP is a constant cycle of audit, reflection and education for continuous quality improvement of the care practice [22]. Audit, as well as research, is greatly facilitated by the electronic LCP. Because the data are readily available, analysis is comprehensive and fast. The audit presented in this study needed no more than a few effort-hours. Further development could bring automatic auditing based on simple program algorithms for data retrieval and analysis, such as proposed for a learning health system [23].

The electronic LCP takes full advantage of the hospital information system. Data protection and archiving is entirely taken care of. Especially archiving, which can be tedious for paper documents, is efficiently provided by the electronic LCP. Neither archive space nor digitalisation is necessary. Despite of all these advantages experienced locally, sites where medical records are predominantly kept on paper may or may not benefit from implementing an electronic LCP. A recent systematic review and meta-analysis did not find substantial improvements on mortality, length of stay or cost by the use of electronic medical records [29]. However, the effective research based on the interoperable data is likely to give valuable answers to clinical questions [23]. As an example, the importance of routine interdisciplinary assessment of LCP patients was emphasised in an analysis of electronic patient data [19].

First reports show that if implemented correctly, it is common (≥1/100 to <1/10) to discontinue LCP care because patients recover [17,19]. A previous study on the same cohort found that 60% of the patients (9/15) were included in the LCP again at a later date [19]. While in those cases the multi-professional team would just start another paper document, the electronic LCP allows the continuation of the LCP care. However, the version used in this study does not provide for multiple recordings of the team decision to begin the LCP documentation. An improved version may need the provision for multiple LCP starts. Another possible improvement is the implementation of automatic reminders for recurrent tasks (such as multi-professional team assessments).

The electronic LCP was used in a SPCU, but not yet on a generic ward. Now, other hospital wards can draw on the experience of the SPCU team to consider implementation of the computerised integrated care plan.

## Conclusion

While integrated care for end-of-life patients such as the LCP is well established, the electronic LCP is an innovation that greatly improves the delivery of integrated care. The electronic version is feasible and efficient. The electronic LCP has a high uptake in the SPCU and a high degree of recorded items. The integration into the existing hospital information system results in automatic archiving. Data retrieval for auditing and research is simple and fast. Computer programs could provide the data for routine auditing. Because the electronic LCP closely reflects the paper version, the members of the team do not need intensive additional training. The successful implementation of the electronic integrated care instrument indicates that, in general, the delivery of integrated care may benefit from the use of electronic infrastructure.

## Abbreviations

LCP, Liverpool Care Pathway; NA, not applicable; RTS, respiratory tract secretions; SPCU, specialized palliative care unit.
